# Development and Characterization of Polymorphic Microsatellite Markers (SSRs) for an Endemic Plant, *Pseudolarix amabilis* (Nelson) Rehd. (Pinaceae)

**DOI:** 10.3390/molecules20022685

**Published:** 2015-02-04

**Authors:** Qi-Fang Geng, Jun Liu, Lin Sun, Hong Liu, Yan Ou-Yang, Ying Cai, Xin-Sheng Tang, Hong-Wei Zhang, Zhong-Sheng Wang, Shu-Qing An

**Affiliations:** 1 School of Life Science, Nanjing University, Nanjing 210093, China; E-Mails: gengqifang@126.com (Q.-F.G.); liujun.mc@163.com (J.L.); 1988bingshan@126.com (L.S.); oyy0723@163.com (Y.O.-Y.); caiying79@126.com (Y.C.); 2 State Key Laboratory of Pharmaceutical Biotechnology, School of Life Science, Nanjing University, Nanjing 210093, China; 3 International Center for Tropical Botany, Department of Earth and Environment, Florida International University, Miami, FL 33199, USA; E-Mail: hliu@fiu.edu; 4 College of Forestry, Guangxi University, Nanning 530004, China; 5 College of Life and Environment Sciences, Huangshan University, Huangshan 245041, China; E-Mail: xstang@hsu.edu.cn; 6 Administration of Zhejiang Qingliangfeng National Nature Reserve, Lin’an 311321, China; E-Mail: laqlf@163.com

**Keywords:** microsatellite markers, ESTs, SSRs, *Pseudolarix amabilis*, Pinaceae

## Abstract

*Pseudolarix* (Pinaceae) is a vulnerable (sensu IUCN) monotypic genus restricted to southeastern China. To better understand levels of genetic diversity, population structure and gene flow among populations of *P. amabilis*, we developed five compound SSR markers and ten novel polymorphic expressed sequence tags (EST) derived microsatellites. The results showed that all 15 loci were polymorphic with the number of alleles per locus ranging from two to seven. The expected and observed heterozygosities varied from 0.169 to 0.752, and 0.000 to 1.000, respectively. The inbreeding coefficient ranged from −0.833 to 1.000. These markers will contribute to research on genetic diversity and population genetic structure of *P. amabilis*, which in turn will contribute to the species conservation.

## 1. Introduction

*Pseudolarix* is a monotypic genus in the family Pinaceae. The fossil record of *Pseudolarix* reveals wide distribution in North America and Eurasia during the Early Cretaceous (Berriasian), possibly the Late Jurassic (Portlandian) and the Plio-Pleistocene [1]. The only species of this genus, *Pseudolarix amabilis* is threatened, and it is restricted to hills and alluvial plains, commonly below 1000 m in elevation, along the lower Yangtze River valley in southeast China [1]. Its primary habitat is highly fragmented and degraded because of intense and relatively recent human activities [2]. This Chinese endemic species is listed as vulnerable based on the IUCN criteria [2,3]. Comprehensive knowledge on genetic diversity and population structure of natural populations is essential for species management and conservation, as it reflects the status and survival potential of populations. Several studies have focused on the various aspects of the biology of *P. amabilis* [4,5,6,7,8,9,10], including a population genetic study [11]. However, the existing population genetics study of *P. amabilis* was based on dominant molecular markers (*i.e*., RAPDS) and was limited to nine populations from the coastal province of Zhejiang.

In recent years, microsatellites or Simple Sequence Repeats (SSRs) have been widely used in studies of population genetics because of their abundance, high level of polymorphism, co-dominance, bi-parentally inheritance, and easy detection by polymerase chain reaction (PCR) [12,13,14]. In general, SSRs are divided in two categories: (1) genomic SSRs developed from random genomic sequences and (2) EST-derived SSR markers (EST-SSRs) derived from expressed sequences tags in public sequence database [15]. An improved genomic SSR technique, *i.e*., isolation of codominant compound SSR markers from genomic DNA, has been recently developed and applied to population genetics studies in both plant and animal species [13,16,17,18]. In this approach, a locus-specific primer is designed from the sequence flanking the compound SSR, and the primer pairs of the locus-specific and compound SSR primers were used as a compound SSR marker. This approach sidesteps the second step of the dual-suppression-PCR method and enable substantial time and cost savings [19,20].

In this study, we developed both compound SSR markers and EST-derived SSR markers from *P. amabilis* in order to assess its genetic diversity, population genetic structure throughout its distribution, which will likely have important implications for its conservation and management strategies.

## 2. Results and Discussion

For the compound SSR markers, a total of 300 positive clones were chosen and tested. We randomly chose 95 fragments to sequence, of which 86 were successfully sequenced, six had the same sequence. All successfully sequenced fragments were flanked by a compound SSR sequence at one end. In total, 69 fragments that contained (AC)_6_(TC)_n_ (1), (TC)_6_(AC)_n_ (25), (AC)_6_(AG)_n_ (21), or (TC)_6_(TG)_n_ (22) repeats at one end were used to design the IP1 primers. Of 69 compound microsatellite loci isolated from *P. amabilis*, 30 loci were successfully amplified and yielded a single PCR product each. Five of these loci were polymorphic in the initial pilot studies with one population.

Among the 118 EST-SSRs, 27 (22.9%) were dinucleotide repeats, and 91 (77.1%) were trinucleotides. The most common dinucleotide and trinucleotide repeats were motifs (AT)_n_ (13.8%) and (TAC)_n_ (9.3%). The motifs (AT)_n_, (AC)_n_, (AG)_n_ (TC)_n_ and (GT)_n_ accounted for 22.9% of the 118 random selected EST-derived SSR markers. Thirty five of the primer pairs (29.7%) amplified the expected products. Of the 35 amplified loci, ten (8.5%) loci were polymorphic in the test population.

Details pertinent to PCR amplification features and genetic diversity parameters for these 15 polymorphic microsatellite loci are presented in Table 1. There were two to seven alleles per locus (Table 1). The expected (*H*_E_) and observed (*H*_O_) heterozygosities ranged from 0.169 to 0.752, and from 0.000 to 1.000, respectively (Table 1). The inbreeding coefficient (*F*_IS_) ranged from −0.833 to 1.000. Seven loci deviated significantly from Hardy Weinberg Equilibrium (*p <* 0.001) (Table 1), because of an excess of homozygotes. Null alleles, inbreeding, Wahlund effect, and small population size might lead to the excess of homozygotes. Because it is usually difficult to avoid null alleles, we recommend that the future population genetic studies of *P. amabilis* correct the genotype data from these 15 microsatellite loci by software (e.g., MICRO-CHECKER [21]). Moreover, the effects of sample and habitat size also should be determined in the population genetic studies. Among the 15 microsatellite loci, significant linkage disequilibrium was detected between PA67 and PA89 (*p* < 0.001). With the compound SSR and EST-SSR markers developed in our study, further studies can be conducted. These SSR markers should be useful to investigate the genetic diversity, population genetic structure, and gene flow of *P. amabilis*.

## 3. Experimental Section

### 3.1. Plant Materials and DNA Extraction

A total of 24 individuals of *P. amabilis* were collected from a single population located in Changxing County, Huzhou, China (31°7'28''N, 119°45'22''E). Leaves were randomly sampled from trees at an interval of at least 10 m and immediately put into the plastic sealed bag with silica gel for fast drying, and then stored at room temperature until use. Total genomic DNA of *P. amabilis* was extracted from dried leaves using a modified Cetytrimethyl Ammonium Bromide (CTAB) method with minor modifications [22]. The DNA was dissolved in 200 µL of sterilized water and kept at −30 °C until use. Two methods were employed to isolate SSR markers for *P. amabilis*, a technique for isolating codominant compound SSR markers, and expressed sequence tag (EST)-derived SSR markers.

### 3.2. Compound SSR Markers

Genomic DNA of *P. amabilis* was extracted from dried leaves and then was separately digested with six blunt-end restriction enzymes, *i.e*., *Rsa*I, *Alu*I, *Eco*RV, *Hae*III, *Hin*cII and *Ssp*I. The restriction fragments were then ligated with a specific blunt adaptor (consisting of the 48-mer: 5'-GTAATACGACTCACTATAGGGCACGCGTGGTCGACGGCCCGGGCTGGT-3' and an 8-mer with the 3'-end capped with an amino residue: 5'-ACCAGCCC-NH_2_-3') using the Takara DNA ligation kit.

**Table 1:** Characteristics of 15 microsatellite markers isolated from *Pseudolarix amabilis*.

Locus	Primer Sequence (5'-3')	Modif	GenBank Accession No.	*T*_a_ (°C)	No. of Samples	Size Range (bp)	No. of Alleles	*H*_O_	*H*_E_	*F*_IS_
Psam05	R: CTAGTGCATCGGGTTGCTAAA	(AC)_6_(AG)_22_	KP418960	54	24	139–147	4	0.417	0.600	0.325
	F: ACACACACACACACAGAGAG									
Psam06	R: GGATTGTAGTCATTGGAGTTAGAG	(AC)_6_(AG)_5_...(TC)_20_	KP418961	53	24	226–232	4	0.579	0.680	0.175
	F: ACACACACACACACAGAGAG									
Psam09 *	F: CCAAGGTCTGATGTAGATTGCTTG	(AC)_6_(AG)_20_	KP418962	58	24	140–148	5	0.917	0.675	−0.339
	F: ACACACACACACACAGAGAG									
Psam44 *	R: CGTCAATGATCCATAATGGCTACA	(AC)_6_(AG)_30_	KP418963	56	24	294–306	7	0.391	0.752	0.497
	F: ACACACACACACACAGAGAG									
Psam45 *	R: TACAATCTCAAGTAGGTCCGTAT	(AC)_6_(AG)_25_	KP418964	55	24	169–183	5	0.042	0.445	0.910
	F: ACACACACACACACAGAGAG									
PA09	F: U19-CGTAGTATGAGGACCACAGTT	(GAA)_9_	SRR290437.4323.2	55	24	161–167	3	0.333	0.500	0.412
	R: TTCTGCATCCTACAAGGTTT									
PA25	F: U19-AATGGGCATTGAACTTCTTA	(AC)_19_	SRR290437.17687.2	54	24	245–247	2	0.000	0.320	1.000
	R: GACGCGACCACACCTAT									
PA51	F: U19-CAAATACAATTACGCCTTCG	(AT)_11_	SRR290437.68993.2	55	24	138–140	2	0.000	0.408	1.000
	R: GTCCACTGTCCAACTTTGTT									
PA60 *	F: U19-AAGATCATGGAGAATTGCTG	(AAG)_12_	SRR290437.29511.2	56	24	191–195	3	0.000	0.169	1.000
	R:AGCTTGAGGTCACGATAGAA									
PA67	F: U19-AAACATGATGGAGGAATACG	(TCT)_7_	SRR290437.39562.2	56	24	132–135	3	0.045	0.208	0.790
	R:TTGAAGGAAACAGAAACAGC									
PA78 *	F:U19-ATGGAAGGAGGATGGTAAAT	(GAG)_8_	SRR290437.49150.2	56	24	263–275	6	1.000	0.650	−0.520
	R:AAACTCTTCCATGAGAGCAA									
PA84	F: U19-ACCATTCTATCGTTTCTCCA	(GCA)_8_	SRR290437.51696.2	56	24	225–239	3	0.158	0.314	0.518
	R:GCCACATGAGCAGAAGACTA									
PA89 *	F: U19-TATTGCTAGAAGAGGGCAAG	(TTC)_8_	SRR290437.57471.2	56	24	299–307	3	0.042	0.414	0.903
	R:CTTGTAGTAGGTGCGGAATC									
PA93	F: U19-CATCCCGTTCTTGATCTTAC	(CTG)_8_	SRR290437.62539.2	56	24	275–284	2	0.158	0.494	0.695
	R:GAAACTTAGGGTTTGGAAGG									
PA107 *	F: U19-TCTCCGGCACTAGTAGACAT	(CAG)_7_	SRR290437.67270.2	56	24	198–205	4	1.000	0.543	−0.833
	R:TGGTCAGTTTGATGAGAACA									

*T*_a_, annealing temperature of the primer pair; * indicates significant deviation from Hardy-Weinberg equilibrium (*p* < 0.001).

Fragments flanked by an SSR region at one end were amplified by PCR from the *Eco*RV and *Ssp*I DNA library using a compound SSR primer (AC)_6_(TC)_5_, (TC)_6_(AC)_5_, (AC)_6_(AG)_5_, (AG)_6_(AC)_5_ or (TC)_6_(TG)_5_ and an adaptor primer (5'-CTATAGGGCACGCGTGGT-3'). The amplified fragments were cloned into Trans^TM^ *pEASY*-T1 Cloning vector system (Transgen, Beijing, China). Transformants were identified by blue/white screening on LB agar plates containing Ampicillin, X-gal and IPTG. The cloned fragments were amplified using the M13 forward and reverse primers from the plasmid DNA of positive clones. The plasmid DNA of the fragments (from 400 to 550 bp) were sequenced using M13 primer by Invitrogen. For each fragment containing an (AC)_6_(TC)*_n_*, (TC)_6_(AC)*_n_*, (AC)_6_(AG)*_n_*, (AG)_6_(AC)*_n_* or (TC)_6_(TG)*_n_* compound SSR sequence at one end, a locus-specific primer (IP1) was designed from the region flanking the compound SSR sequence. The IP1 and corresponding compound SSR primers were used as a compound SSR marker.

### 3.3. Expressed Sequence Tag (EST) SSR Markers

Transcriptome sequences of *P. amabilis* were downloaded from the Short Read Archive (SRA) of the National Center for Biotechnology Information (NCBI) with accession numbers of SRR290437 (http://www.ncbi.nlm.nih.gov/sra/?term=pseudolarix+amabilis). A total of 733,434 unigenes with a total length of 855.4 Mb were obtained. The obtained unigenes were then analyzed using the SciRoKo v3.4 software [23] to identify reads containing simple tandem repeats. We used the Mismatched; Fixed Penalty mode with a mismatch penalty of five to identify microsatellites. At least 10 repeats for dinucleotide motifs and six repeats for trinucleotide motifs were selected for further study. We designed the primers using PRIMER 3 software [24] with the following parameters, a 40%–60% GC content, 100–300 bp final product size, primer T_m_s of 52–58 °C (optimum 55 °C) and a 3 °C maximum difference in melting temperature between paired primers. Primers were successfully designed for 12,141 of these microsatellite-contained sequences. We randomly selected 118 of the 12,141 primer pairs for PCR validation.

### 3.4. Polymorphism Detection

PCR amplification was carried out with a reaction mixture (10 μL), containing about 10 ng of template DNA, 1× AmpliTaq Gold 360 Master Mix (Applied Biosystems, Carlsbad, CA, USA), 4% of 360 GC Enhancer (Applied Biosystems) and 0.5 μM of each designed primer.

For the loci isolated by the compound SSR method, the corresponding markers (*i.e*., (AC)_7_(TC)_3_, (TC)_7_(AC)_3_, (AC)_7_(AG)_3_ or (TC)_7_(TG)_3_) were labeled with fluorochromes 6-FAM, VIC, NED or PET (Applied Biosystems).

For the EST-derived SSR markers, we used a tailed primer method to perform PCR, a U19 (5'-GGTTTTCCCAGTCACGACG-3', [25]) was tailed to the 5' end of the forward primer, and the U19 primer labeled with 6-FAM, VIC, NED or PET, was added to the PCR reaction mix.

The PCR reactions were conducted on a Veriti Thermal Cycler (Applied Biosystems). The PCR reaction was performed using the following cycling conditions: denaturation at 95 °C for 10 min; followed by 38 cycles of 30 s at 95 °C, 30 s at annealing temperature of primer pair and 1 min at 72 °C; then final extension at 72 °C for 7 min. PCR products were analyzed using an ABI 3730 genetic analyzer (Applied Biosystems). Alleles sizes were determined using the GeneMapper^TM^ analysis software version 4.0 (Applied Biosystems) according to a LIZ-500 DNA size standard (Applied Biosystems).

### 3.5. Data Analysis

Number of alleles per locus (*N*_A_), observed heterozygosity (*H*_O_), and expected heterozygosity (*H*_E_) were assessed using GENALEX 6.5 [26,27]. Hardy-Weinberg equilibrium (HWE) and linkage disequilibrium between pairs of microsatellites were calculated with Genepop 4.2 [28,29]. The inbreeding coefficient (*F*_IS_) was calculated by FSTAT version 2.9.3 [28].

## 4. Conclusions

We successfully developed and characterized SSR markers derived from the expressed sequence tags as well as codominant compound SSRs isolated from genomic DNA of *P. amabilis*. With these fifteen polymorphic SSR markers, further population genetic studies of *P. amabilis* can be carried out for the species’ conservation and management.
